# Case of Extra Uterine Fœtation

**Published:** 1872-03

**Authors:** Harvey Jewett

**Affiliations:** Canandaigua, N. Y.


ART. II.—*Case of Extra Uterine Fœtation,* By HARVEY JEWETT M. D., Canandaigua, N. Y.

A paper read before the State Medical Society at their meeting in February, 1872.

Extra Uterine Fœtation in many of its forms is of such rare occurrence, especially in private practice, that the following case of tubal pregnancy is deemed worthy of record.

Perhaps, if in all the instances of sudden death, a more scrutinizing investigation as to the cause, could be instituted, many cases that are now shrouded in mystery might reveal numerous instances of this abnormal condition.

Mrs. F., the wife of a farmer, of good constitution, had been married 16 years without offspring. During the months of May and June, 1856, she found herself unusually indisposed and applied to her family physician, who assured her that she was pregnant. On the 8th of August she consulted the late Dr. E. W. Cheny, to whom I am indebted for the leading facts in the history of her case, and by whose courtesy I was permitted to examine her in the last stage of her illness and make the examination after her death.

The patient stated that in jumping from a buggy to the ground a few days previously she was severely jarred inwardly, pointing to the right Illiac region; but was not entirely disabled from her domestic duties. She was seized next morning, while at the breakfast table with severe pains in the lower part of the bowels, followed by exhaustion and faintness.

Dr. C. was immediately called and found her in great pain in the uterine region, small, rapid pulse, sallow, exsanguineous countenance, and the whole expression of her face betrayed the most intense anxiety and suffering. The abdomen was considerably distended, which was supposed to be partly chargeable to retention of urine, and constipated bowels. Accordingly, a catheter was introduced into the bladder, and the bowels unloaded by a cathartic, with a slight dimunition of the fullness.

The pain, bloating and prostration continued, notwithstanding the most vigorous use of anodynes and stimulants, until the third day when she sunk down from exhaustion and died.

The *post mortem* 24 hours after death, revealed the mystery hanging over the cause of death. The peritoneal sac was distended with bloody serum, and a large coagula was found in the pelvic cavity. On removing the fluid a fœtus, apparently between three and four months, was found in the Illiac region, having escaped from a rupture in the right fallopian tube near its center, the placental mass was partially attached and remaining in the sac which contained the fœtus.

Cases of Extra Uterine pregnancy are somewhat rare in the human species, and are said never to have occurred in the lower order of animals.

Ramsbotham, and many other writers, divide these abnormal cases in four species, viz. : the ovarian, tubal, abdominal and parietal.

Cazeaux has subdivided these into ten varieties, which have received different names according to the part of the passage where the ovule is arrested and develops.

There is a general concurrence however on the part of all writers, that the tubal is by far the most common of all the forms of extra uterine fœtation. And the reasons are obvious why this should be so, if we but look for a moment at the length and narrowness of the canal through which the fecundated ovule makes its transit from the ovarian bodies to the uterine cavity.

This arrest and developement may take place at any point along this canal wherever it meets with any obstruction, and where it forms adhesions to the inner surface of the tube itself.

Under such circumstances the fibers of the fallopian tube become the enveloping structure of the embryo fœtus, which, by its continued growth distends enormously this structure until it culminates in a rupture of its walls, ordinarily between the third and fourth months.

Instances occur in which the impregnated ovule is not detached from its bed, and the ovarian structure is converted into a sac for the newly organized being.

In this case, which is denominated ovarian pregnancy, the structures are better adapted to development and distention, and the work may go on even to the full period of gestation, and the patient unconscious of her true condition and her eminent peril.

The structures of the walls of the cyst therefore differ according to the place where the fecundated ovule finds a lodgement.

In the tubal variety they consist of the walls of the tube itself and in the ovarian of the integuments of the ovary and its peritoneal envelope.

When such unfortunate conceptions take place, the patient will regard herself pregnant since all the rational signs of pregnancy are present such as cessation of the menses, sickness of the stomach, enlargement of the breasts, and a deeper tinted areola.

Neither can we determine, by the most critical examination, the exact condition and impending danger that threatens the life of our patient. We may determine by exploration that the uterus is unimpregnated, but our diagnosis must be negative, aud conjectural in character. If we find all the rational signs prominently marked and the uterus unimpregnated, we may, with great confidence, *infer* that one of the several forms of extra uterine fœtation above alluded to may exist.

If the patient complains of pain or discomfort during any one of the forms of extra uterine pregnancy, we are not likely to attribute her indisposition to the true cause. We are assured by the best authority that there are no diagnostic indications that characterize unmistakably the peril that awaits the unfortunate victim.

The first indications of danger will often be an instantaneous rupture of the sac, followed by intense pain, exhausting hœmorrhage, palid face, rapid pulse, and all the indications of speedy dissolution.

We are told that under such circumstances we are left to the painful alternative of soothing the suffering, the cause of which is entirely beyond our control.

Numerous instances are on record in our text books on obstetrical practice, in which the subject goes on to the full period of gestation, and even many months and years beyond that period, and the cyst remains intact. Then again, natural labor-pains come on at or about the completion of the ninth month, in some instances resulting in a rupture of the sac with all its disasterous consequences.

Many instances are recorded where the fœtus dies and remains incased in its covering, an innocuous mass within the cavity of the abdomen—the vitality of the cyst in such cases is preserved and the contents remain undisturbed without compromising the life of the mother.

Generally, we are told, however, that the development of the cyst ceases with the life of the fœtus—the circulation becomes more feeble, the parts atrophied and remain in that condition until adhesive inflammation is set up and cements the walls of the cyst and the adjacent parts, ulceration follows and a communication is established with the rectum, colon, or any other organ with which it happens to approximate at the time, and the diseased mass is thrown off through these channels.

An instance of this kind (a patient of the late Dr. Edson Carr,) came under my observation a few years since, in which the bones and putrifying debris of an infant, at the full period, were removed with great difficulty from the rectum.

The mother in this case recovered after a long period of suffering from the pyemia and consequent exhaustion, resulting from the presence and removal of the diseased mass.

In view of the conservative efforts of nature, in rare instances, to cast off this foreign body, we may very naturally and profitably inquire can there be any surgical interference that will afford a reasonable chance of deliverance from the impending results.

There was a time when Ovariotomy was deemed so hazardous that patients were allowed to sink down and die rather than submit to the chances of an operation. But now recovery is the rule, after the removal of ovarian tumors, and success follows the use of the knife in hundreds of instances all over the land.

If the diagnosis can be satisfactorily established in a case of extra Uterine fœtation, I hold we are warranted in giving the patient the benefit of a chance attending an operation for its removal.

A case of this kind I find recorded in the British Medical Journal, in which Prof. McCulloch, of Montreal, removed a child from the abdomen by the Cæsarean section and the mother recovered.

The above well authenticated case sets forth the fact, notwithstanding the protestations of surgical interference by the authors of our American and Continental text books on this subject; and I hold that Gastrotomy is not only justifiable, but it is the bounden duty of the surgeon, when the diagnosis is clear, either in the rupture of a fœtal cyst, or in pelvic hæmatocele to operate at once, remove the accumulated blood, and tie the bleeding vessels.

Dr. Stephen Rogers, of New York, has written an elaborate memoir upon this subject, in which he claims that in all cases of extra uterine pregnancy there is an intra uterine decidua cast off, attended with slight hemorrhage, at some period varying from six weeks to several months, and is often mistaken by the patient for a return of the menstrual period.

This extrusion or expulsion of the decidua is regarded by Dr. Rogers, taken in connection with the other signs spoken of, as a pathognomonic indication of this abnormal condition.

The only pathological condition liable to be mistaken for a rupture of a fœtal cyst is pelvic hæmatocele, which, in some of its leading characteristics bears some analogy to the former disease. But a careful scrutiny into the history and antecedents of the case, will reveal the differential diagnosis so decidedly as to settle the question in the mind of any acute observer.

I would refer those who wish to make a more critical analysis of the subject to a statement published by Dr. Rogers, of New York, in the Medical Record for April, 1868, in which he has set forth in a tabular form, the differential indications that mark these anomalous conditions.

